# Estrogen Receptor Alpha Contributes to Intestinal Inflammation in a Murine Model of Ileitis

**DOI:** 10.33696/immunology.7.232

**Published:** 2025

**Authors:** Alyssia V. Broncano, Wendy A. Goodman

**Affiliations:** 1 Department of Pathology, Case Western Reserve University School of Medicine, Cleveland, Ohio, USA

**Keywords:** Estrogen, Estrogen receptor alpha, Estrogen receptor beta, Inflammatory bowel disease, Crohn’s disease, Inflammation, SAMP ileitis

## Introduction

Inflammatory bowel diseases (IBD) are inflammatory conditions characterized by chronic and recurrent intestinal inflammation [1,2]. IBD affects nearly five million people worldwide, with an estimated prevalence of 245.3 cases per 100,000 people in the United States alone [3]. The incidence of IBD is steadily increasing in industrialized countries and is appearing more often developing countries, contributing to its rise as a global disease burden [4,5].

IBD encompasses ulcerative colitis (UC), in which ulcers and inflammatory patches are localized to the innermost lining of the colon [1], and Crohn’s disease (CD), which has discontinuous patches of inflammation anywhere in the gastrointestinal tract [1]. IBD patients experience symptoms that range in severity, including fatigue, weight loss, blood in stool, and abdominal pain [1]. These symptoms can be debilitating and lead to life-threatening complications, thus impeding quality of life. Symptoms of IBD are observed at all ages, including in childhood. Pediatric IBD patients experience impaired growth development, primarily seen as reduced height and weight measurements compared to healthy peers [6,7]. Nearly 1 out of 4 IBD patients are diagnosed before 18 years of age, and one quarter of affected adolescents are under 10 years of age when diagnosed [8].

Although the exact etiology of IBD is not conclusive, there are numerous factors that are believed to be involved, including genetic predisposition, environmental exposure, and lifestyle [9,10]. Combinations of these factors can lead to physiological changes causing dysregulation of immune responses, mucosal barrier function, and gastrointestinal microbiota composition [11]. Because the causes driving IBD are not fully understood and there is no known cure for IBD, patients turn to costly treatments for managing symptoms. These treatments result in healthcare costs of nearly $23,000 per year [12], with costs driven by treatment of comorbidities (ie. anemia, mental health), emergency room visits, and pharmacotherapies [12,13]. Many treatments use pharmacological intervention to modulate immune responses, primarily by reducing lymphocyte effector function, reducing circulating levels of pro-inflammatory cytokines, and promoting regulatory T cell (Treg) function [14].

17β-estradiol (estrogen, E2) regulates numerous cellular processes, including proliferation, angiogenesis, and inhibition of apoptosis [15]. E2 generally has an immunoprotective role, in which E2 signaling promotes pathogen clearance and tissue repair [16], in part by promoting differentiation of anti-inflammatory immune cell populations including M2 macrophages [17], Th2 cells [18], and Tregs [19]. E2 signals through the estrogen receptors alpha and beta (ERα and ERβ), which are part of the nuclear receptor family [20]. Upon ligand binding, ERs either homo- or hetero-dimerize and translocate to the nucleus, where they modulate transcriptional changes in target genes [21,22]. ERs can bind directly or indirectly (via recruitment of transcriptional coactivators) to estrogen response elements (EREs) on promoter regions of target genes [23,24]. ERα is known to have more pro-inflammatory and pro-proliferative roles, whereas ERβ has been shown to confer a protective role that limits inflammation [25,26].

Dysregulated E2 signaling contributes to progression of inflammatory conditions, such as IBD [14,27]. Patients with active IBD (both UC and CD) have reduced ERβ expression in inflamed colonic mucosal tissues [28–30]. Additionally, previous work from our lab demonstrated that peripheral Tregs from female patients with active CD have significantly reduced expression of ERβ [26]. Together, these suggest that ERβ is important for regulating inflammation in the intestine, and that the normally protective role of ERβ as an “inflammatory brake” is impaired in IBD, especially in female patients. This aligns with other clinical observations of IBD, including that disease incidence of CD is higher in adult women than men and that women are more likely to experience more severe disease manifestations [31]. Several reports have suggested that hormones are likely to play a role in the development of disease [32,33], making E2 a promising target for investigating IBD.

Various animal experimental models have been generated to investigate the mechanisms driving intestinal inflammation. Experimental models of IBD have been used to characterize defects in epithelial barrier integrity, innate immune responses, and adaptive immune responses [34]. The SAMP/YitFC (“SAMP”) mouse model is one such model, which arose spontaneously from a strain of senescence accelerated mice [35] and exhibit Crohn’s-like ileitis that mimics the pathology and female sex bias of human CD [36]. Female SAMP mice display earlier onset and increased severity of ileitis compared to male SAMP [27]. Acute and chronic inflammation are localized to the terminal ileum, where inflammation is transmural and has a discontinuous pattern [36]. Disruption of the epithelial barrier is present, along with crypt elongation and tissue atrophy [37]. Histological analyses show infiltration of various cell types, including lymphocytes, neutrophils, and macrophages [37]. SAMP mice exhibit high IFNγ production by 4 weeks of age [38] and full onset of ileitis by 10 weeks of age [39]. Inflammation progressively worsens as mice age, and by 40–50 weeks they display thickening of the bowel wall and stricture formation in the intestine [39]. While the SAMP model has advantages for modeling chronic and spontaneous IBD, the mechanisms driving disease remain to be fully understood.

Our previous work identified a mechanism by which E2 modulates Treg differentiation and function in SAMP mice. Female SAMP mice with global deletion of ERβ (SAMP^ΔERβ^) exhibited decreased Foxp3 expression in CD4^+^ T cells from the mesenteric lymph node (mLN), along with altered expression of canonical Treg-associated genes from full thickness ileal tissues, and decreased Treg differentiation and suppressive function [26]. These results were observed in female mice but not males, indicating that deletion of ERβ–thus promoting ERα-specific signaling–has a more profound effect in driving intestinal inflammation in females. Here, we assessed whether ERα would also display sex-specific effects in chronic intestinal inflammation using a similar experimental SAMP model. We hypothesized that eliminating ERα-specific signaling, which is well-known for its pro-inflammatory roles, would reduce the severity of ileitis.

## Materials and Methods

Heterozygous ERα^+/−^ mice were back-crossed with SAMP/YitFc mice for eight generations to generate SAMP mice heterozygous for ERα. These heterozygous mice were then bred to generate SAMP mice lacking global expression of ERα (SAMP-ERα-KO), similar to our previously described work in generating SAMP^ΔERβ^ mice [26]. Heterozygous mice were used for breeding because mice with ablation of ERα have impaired fertility [40]. Deletion of ERα was confirmed by Western blot. Male and female SAMP-ERα-KO mice were euthanized at 6, 10, 15, and 20 weeks of age and assessed for ileal inflammation. Total inflammatory scores (TIS) were assigned to each mouse based on a set of metrics determined by histological assessment of the degree of ulceration, re-epithelialization, active inflammation, chronic inflammation, and transmural inflammation, calculated by a pathologist blinded to mouse genotype and sex, as previously described [27].

## Results

**Figure 1.**
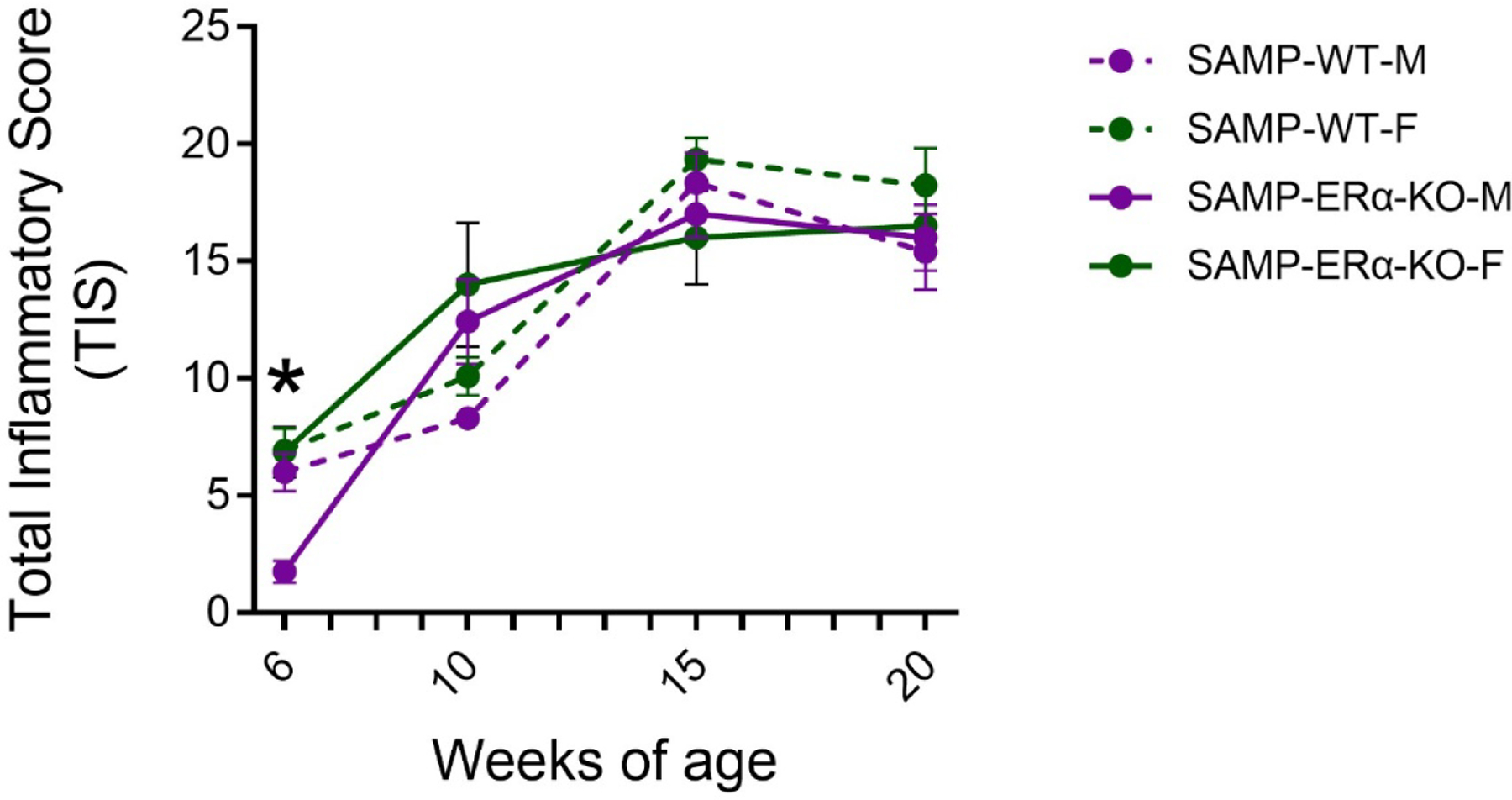
Total inflammatory scores (TIS) from ileal tissues of native SAMP (“SAMP-WT”) and SAMP-ERα-KO mice. Scores were recorded over the course of 6–20 weeks of age. n = 5–13 per age/sex. Data is represented as mean ± SEM. *p<0.01.

To assess the role of ERα on progression of inflammation, ileal tissues were collected from male and female SAMP-ERα-KO mice and evaluated for inflammation by histological staining (H&E). At six weeks of age, female SAMP-ERα-KO mice were found to have significantly higher Total Inflammatory Scores (TIS) compared to males, indicating more severe ileitis (Figure 1). This data complements our previous findings that female native SAMP mice display earlier onset of ileitis compared to males beginning at 6 weeks of age [27]. TIS in native SAMP and SAMP-ERα-KO mice increase dramatically from ages 6 to 10 weeks, but then plateau from ages 10–20 weeks for both males and females [27] (Figure 1). After 10 weeks of age, there are no significant differences in TIS between males and females, demonstrating the loss of female-biased ileitis normally observed in SAMP mice [27]. This suggests that ERα might be necessary to promote ileal inflammation, specifically in females.

## Discussion

IBDs exhibit chronic inflammation in the intestine with high rates of relapse. Although the exact mechanisms causing IBD remain to be elucidated, many reports have suggested that E2 signaling is a strong contributing factor. Our previous work showed that female, but not male, SAMP mice with deletion of ERβ had exacerbated symptoms of intestinal inflammation, suggesting that inflammation is driven by ERα-specific signaling [26]. In agreement with our previous work, the current results demonstrate that deletion of ERα results in a similar degree of inflammation between male and female SAMP mice as inflammation becomes more chronic over time (>6 weeks of age). Indeed, the deletion of ERα eliminates the sex bias of exacerbated inflammation normally present in female SAMP mice. These results also suggest that deletion of ERα–thereby promoting ERβ-specific signaling–may be useful in revealing protective roles of ERβ in regulating SAMP ileitis. This complements our previous findings, in which we found that deletion of ERα is protective in chemically-induced colitis [41].

We hypothesized that deletion of ERα would have a protective effect in SAMP mice by reducing overall severity of inflammation. However, our observations were not entirely as expected. SAMP-ERα-KO mice maintained high TIS that increased with age, similar to development of disease in native SAMP [36], demonstrating that eliminating ERα does not prevent inflammation from occurring. However, the degree of inflammation became similar in males and females (Figure 1). TIS from SAMP-ERα-KO mice showed a female sex bias at 6 weeks of age, around the time of puberty [42]. In native SAMP mice, females show earlier onset of inflammation beginning around 6 weeks and experience more severe inflammation throughout the course of disease [27]. This suggests that disease severity worsens when E2 has increased biological activity and more active signaling. This aligns with other clinical findings where women with autoimmune conditions experience enhanced inflammation during puberty, menstruation, or pregnancy, when E2 levels are higher [43]. Additionally, women receiving hormone treatments such as oral contraceptive pills, which raise serum E2 levels, are correlated with increased risk for IBD and worsened symptoms [44,45]. Because we observe in our model that inflammation is similar in males and females beginning at 10 weeks, we can speculate that the lack of ERα-specific signaling may prevent inflammation from getting worse. Collectively, these results suggest a pathogenic role of ERα in driving inflammation in SAMP mice.

Given the known pro-inflammatory roles of ERα, together with higher levels of circulating E2 in women, we can speculate that it has a more pronounced role in enhancing intestinal inflammation in females than males. We can also speculate that a major role of ERβ is to regulate activity of ERα to modulate inflammation. ERβ is the predominant estrogen receptor expressed in the colon and is highly expressed in the intestine compared to other peripheral tissues [46]. ERβ regulates growth, organization, and maintenance of epithelial structure to protect intestinal epithelial barrier integrity and function [47,48]. ERβ also promotes autophagy, an important process in maintaining intestinal homeostasis, by supporting function of intestinal epithelial cells, regulating microbiota, and modulating immune responses by promoting an anti-inflammatory environment [49]. Autophagy is dysregulated in both IBD patients and experimental IBD, and treatment with autophagy agonists has been shown to improve intestinal inflammation [50].

Significant reduction of ERβ expression has been observed in both experimental IBD and in IBD patients [46,51]. Interestingly, decreased ERβ expression is only observed in patients with active IBD, as levels of ERβ are unchanged for individuals in remission [28]. This suggests that relative lack of ERβ signaling (or enhanced ERα signaling) is associated with active inflammation. Restoring ERβ expression and promoting ERβ-specific signaling has been shown to alleviate DSS-induced colitis in mice [29]. Specifically, activation of ERβ resulted in reduced weight loss, lower levels of pro-inflammatory cytokines, and reduced histopathological damage [29]. It is possible that the SAMP-ERα-KO disease model used for our study exhibits improved intestinal epithelial integrity contributing to less severe inflammation in females, but further investigation is needed to better elucidate the mechanisms involved.

Our findings show that diminished ERα-specific signaling (which leads to enhanced ERβ-specific signaling) is beneficial for reducing inflammation in IBD. It is likely in IBD that ERα signaling is unchecked by ERβ, and this lack of regulation enables ERα to promote proinflammatory, mitogenic signaling pathways. Since ERβ expression is reduced in IBD, it could be beneficial for therapies to focus on raising endogenous ERβ expression in the intestine or promoting ERβ-specific signaling. ERβ has been shown to reduce collagen deposition via the TGFβ and TLR4 signaling pathways, therefore alleviating intestinal fibrosis [30], thus making it an attractive target for anti-inflammatory treatment. Additionally, IBD has T cell-driven components that may be mediated by E2 signaling activity. Studies have reported that high levels of E2 induce T cell differentiation and that these generated T cells are thought to be more autoreactive and contribute to higher incidence of autoimmune disease in females [52]. Males, on the other hand, have higher frequencies of Tregs than females, in part due to elevated androgens and decreased ERα signaling [53]. Because estrogen levels are different in males and females, it is important to carefully devise therapies that can promote recovery without many off-target effects. More research is needed to better understand the nuances of ERα- versus ERβ-specific signaling and their roles in inflammatory processes.

## Abbreviations

IBD: Inflammatory Bowel Disease
UC: Ulcerative Colitis
CD: Crohn’s Disease
E2: 17β-estradiol, Estrogen
Erα: Estrogen Receptor Alpha
Erβ: Estrogen Receptor Beta
Treg: Regulatory T cell
SAMP: SAMP/YitFC
